# Effect of the Addition of Whole and Milled Flaxseed on the Quality Characteristics of Yogurt

**DOI:** 10.3390/foods10092140

**Published:** 2021-09-10

**Authors:** Patrycja Cichońska, Ewelina Pudło, Adrian Wojtczak, Małgorzata Ziarno

**Affiliations:** 1 Department of Food Technology and Assessment, Institute of Food Science, Warsaw University of Life Sciences-SGGW (WULS-SGGW), 02-787 Warsaw, Poland; ewelina_agnieszka_pudlo@wp.pl (E.P.); malgorzata_ziarno@sggw.edu.pl (M.Z.); 2 Department of Microbiology, Prof. Waclaw Dabrowski Institute of Agriculture and Food Biotechnology, 02-532 Warsaw, Poland; adrian.wojtczak@ibprs.pl

**Keywords:** functional food, milk fermentation, flaxseed, active acidity, yogurt bacteria, apparent viscosity, syneresis, bioactive compounds

## Abstract

The present study aimed to analyze the effect of the addition of whole and milled flaxseed on the quality characteristics of yogurt. In the first stage of the research, the optimal dose of flaxseed was determined. In the second stage of the research, it was assessed whether the selected qualities of yogurt were affected by the form of flaxseed (whole or milled) and the time of addition (before or after fermentation). The yogurts obtained were stored at 5 °C for 21 days, and the changes in active acidity, apparent viscosity, syneresis, and the number of yogurt bacteria were determined. The results of the second stage of the study were subjected to two-way analysis of variance (ANOVA) (*p* < 0.05). The study showed that the addition of milled flaxseed to yogurts in the amount of 1% was optimal. Time and form of flaxseed supplementation significantly influenced the changes in active acidity, apparent viscosity, and syneresis in the tested yogurts. The addition of flaxseed did not significantly change the content of yogurt bacteria. The results indicate that to achieve increased apparent viscosity and reduced syneresis, it is more advantageous to use milled flaxseed rather than whole flaxseed.

## 1. Introduction

Consumers’ awareness of the importance of a healthy diet is growing and, therefore, they often prefer products containing functional foods [1,2]. Functional foods can be defined as foods or food ingredients that can provide physiological benefits and help in the prevention and/or treatment of disease [3]. The dairy industry is increasingly using various types of functional ingredients as additives to traditional products such as yogurt. This allows not only a new range of products to be created but also their properties to be modified by limiting the use of food additives [4,5]. Flaxseed is an interesting additive which not only affects the nutritional value of the product but also improves its structure by influencing its rheological properties [3].

Yogurt is one of the most popular fermented dairy products worldwide that has great consumer acceptability because of its health benefits [6]. The basic ingredients of yogurt are milk and live yogurt bacteria cultures [7]. The active strains of bacteria are mainly *Streptococcus thermophilus* and *Lactobacillus delbrueckii* subsp. *bulgaricus* [8,9]. Consumption of yogurt has a positive effect on health as it contributes to the prevention of gastrointestinal infections, lowering of serum cholesterol level, and antimutagenic activity [6]. Lactic acid bacteria (LAB) help to increase the absorption of nutrients, participate in the synthesis of vitamins, and reduce the development of undesirable microflora in the intestine [7,8,10]. Because of these known health benefits of yogurt, the consumer demand for yogurt has increased. Yogurts are now manufactured in several styles and varieties with different fat contents, flavors, and textures [6].

During the production of yogurt, it is important to obtain its appropriate quality characteristics, which include acidity, flavor characteristics, clot structure, and degree of syneresis. The quality characteristics of yogurt can be controlled and improved using stabilizers, e.g., starch, guar gum, carboxymethyl cellulose, carrageenan, and pectin [11,12,13]. The use of stabilizers allows us to modify rheological properties (mainly viscosity) and reduce the level of syneresis [13,14]. The “clean label” trend popular among consumers encourages producers to use natural plant materials for stabilization purposes, the presence of which in the product does not raise any controversies or health concerns [15].

Flaxseed (*Linum usitatissimum* L.), also known as linseed, is one of the most important oilseed crops for industrial applications [16,17]. It produces small, flat seeds whose color range from golden yellow to reddish brown [18]. It is used as food and animal feed and to produce fiber. As a food, flaxseed is used in the form of whole seeds, flour, or as oil [19,20]. The pro-health effect after consuming flaxseed depends on the type of product and form of consumption of the seed [21]. Flaxseed is a rich source of proteins (10–30% of the content), fat (40% of the content), and fiber (28% of the content) [16,22,23,24,25]. Food fortification with flaxseed components has been proven to offer many health benefits [26,27]. Flaxseed is considered as a functional food because of the high content of bioactive ingredients such as α-linolenic acid (ALA), lignans, phenolic compounds, and soluble fiber [18,20,28]. Previous studies have proved that flaxseed has a potential in disease prevention, particularly cardiovascular disease (CVD), obesity, osteoporosis, rheumatoid arthritis, and breast and colon cancer, and can boost immunity [24,25,27,29]. Lignans are a phytoestrogen that helps in decreasing cell proliferation and can prevent cancer [16,17,20]. ALA is beneficial for infant brain development and for reducing blood lipid levels, and it has anti-inflammatory, anticoagulant, and antiarrhythmic properties [3,18]. The soluble fiber fraction contains mucilaginous substances (5–8% of the soluble fraction), which are characterized by high water absorption and functional properties like those of Arabic gum. Consuming soluble fiber helps to prevent constipation, lowers the risk of colon and rectal cancer, lowers total cholesterol level, and regulates blood sugar fluctuations [24,25,26,27]. Flaxseed also contains antinutritional components, including cyanogenic glycosides, cadmium, and trypsin inhibitors. Their presence should be reduced or eliminated as much as possible with mechanical and thermal processing [20,21,22].

Because of the high content of proteins, fiber, and mucous substances, flaxseeds are used for producing new food products [25]. To date, flaxseed has been used in different forms for the production of various dietary products such as baked cereal products, fiber bars, meat fillers, bread, muffins, and spaghetti [3]. The enrichment of food products with flaxseed affects the sensory characteristics, nutritional value, and rheological properties of the products [30]. In the technology of dairy products, the most important component of flaxseed is soluble fiber, which may have a stabilizing effect. The key issues when using various stabilizers in dairy formulations are improvement of their physical properties, including syneresis, gelling, thickening capacity, stability, and rheological properties [31,32]. Soluble dietary fiber-enriched dairy products may serve as functional foods, as dietary fiber helps to improve sensory characteristics, shelf life, and structural properties (i.e., viscosity, texture, and water and oil holding capacity) of dairy foods [26,27].

Flaxseed is emerging as one of the nutritive and functional ingredients in food products. [25] It increases the availability of healthy food and enhances the nutrient profile of foods by reducing salt, sugar, and saturated fat content as well as by increasing the content of bioactive compounds. Markets for healthy foods are growing worldwide, and in the future, flaxseed can be used as an ingredient of functional foods and nutraceuticals, which are foods with health-promoting or disease preventive properties [29,33]. The functional and health-promoting properties of flaxseed can enable the food processing industry to create a large variety of foods that contain this plant, thus offering the public an increased opportunity to incorporate flaxseed into their everyday diet. In the coming years, it is expected that flaxseed will become more economically important for farmers, food processers, and retailers in food market [34]. Therefore, the present study aimed to analyze the effect of the addition of whole and milled flaxseed on the quality characteristics of yogurt. The influence of the addition of flaxseed in various forms and at various stages of yogurt production on the selected quality characteristics of the product during the storage period was investigated.

## 2. Materials and Methods

### 2.1. Materials

The research materials were ultra-high temperature (UHT)-treated milk (Mlekovita, Wysokie Mazowieckie, Poland, 2.0% fat content) for producing yogurts; gold flaxseed (Agnex, Białystok, Poland), milled flaxseed (Agnex, Białystok, Poland), and freeze-dried yogurt culture YC-X16 (Chr. Hansen, Warsaw, Poland, containing *Streptococcus thermophilus* and *Lactobacillus delbrueckii* subsp. *bulgaricus*).

### 2.2. Methods

The research was performed in two stages. In the first stage (pilot study), the optimal dose of flaxseed was determined, the addition of which met the physicochemical and rheological parameters of the yogurt. At this stage, milled flaxseed in the amount of 1%, 2%, or 3% relative to the amount of milk was added to the UHT-treated milk, except for the control sample, and the whole mixture was pasteurized at 80 °C for 5 min. All yogurt variants (0, 1%, 2% and 3% flaxseed supplement) were prepared in nine samples in jars of 160 mL each. The samples were then cooled to 39 °C and inoculated with 0.004% yogurt bacteria starter culture. The inoculated and mixed solutions were incubated at 37 °C for 5 h. After the incubation period, the yogurts were cooled and stored at 5 °C for 24 h. After this time, the pH, apparent viscosity, and syneresis of the obtained yogurts were analyzed.

In the second stage of the research (main research), four types of yogurt were prepared with the addition of an optimized dose of flaxseed, which was determined in the first stage of the research. All yogurt variants were prepared in 12 samples in jars of 160 mL each. All samples were prepared by inoculating UHT-treated milk at 39 °C with yogurt bacteria starter culture at the dose of 0.004%. The fermentation process was carried out at 37 °C for 5 h, and the samples were then cooled to 5 °C. Sterilized (121 °C/5 s) whole or milled flaxseed were added to the samples before or after the fermentation process to obtain the following yogurt variants:

- Variant 1: yogurt with the addition of milled flaxseed added before fermentation;
- Variant 2: yogurt with the addition of whole flaxseed added before fermentation;
- Variant 3: yogurt with the addition of milled flaxseed added after fermentation;
- Variant 4: yogurt with the addition of whole flaxseeds added after fermentation;
- Control sample: yogurt without the addition of whole or milled flaxseed.

The yogurts obtained were stored at 5 °C for 21 days. In the prepared samples, the changes in active acidity, apparent viscosity, syneresis, and the number of yogurt bacteria during the storage period were determined.

#### 2.2.1. Active Acidity (pH) Analysis

The active acidity (pH) analysis was performed using the Elmetron CPO-505 pH meter (Elmetron, Zabrze, Poland). The device was properly calibrated using the buffer solutions according to the manufacturer’s instructions. The analyses were performed by immersing the pH meter electrode in the obtained yogurts. Measurements were made in 3 repetitions according to the following measurement points:

For pilot studies (first stage of research):

- after 3 h of incubation at 37 °C.
- after 24 h of storage at 5 °C.

For main research (second stage of research):

- after 24 h of storage at 5 °C.
- after 7 days of storage at 5 °C.
- after 14 days of storage at 5 °C.
- after 21 days of storage at 5 °C.

#### 2.2.2. Apparent Viscosity Analysis

The apparent viscosity of the yogurts was determined using a Brookfield RV-D-II + pro viscometer (Brookfield Engineering Laboratories INC., Middleboro, MA, USA) coupled with a computer software (Brookfield Engineering Laboratories INC., Middleboro, MA, USA). Spindle no. 04 was used for the assessment. The rotation speed was 10 rpm. The spindle was completely immersed in the mixed samples. The analysis was performed at 5 °C. Measurements were taken in three repetitions according to the following measurement points:

For pilot studies (first stage of research):

- after 24 h of storage at 5 °C.

For main research (second stage of research):

- after 24 h of storage at 5 °C.
- after 7 days of storage at 5 °C.
- after 14 days of storage at 5 °C.
- after 21 days of storage at 5 °C.

#### 2.2.3. Syneresis Analysis

Syneresis was determined using the MPW-350R centrifuge (MPW MED. INSTRUMENTS, Warsaw, Poland). The samples were mixed, weighed to 40 g, and centrifuged at 4 °C and 16,125× *g* for 20 min. After completion of centrifugation, the separated whey was decanted and weighed. The value of syneresis *S* [%] was calculated according to Formula (1):

*S* = *A*/*B* × 100%, (1)

where:

*A*—mass of whey separated during centrifugation [g]

*B*—yogurt mass before centrifugation [g]

Measurements were made in three repetitions according to the following measurement points:

For pilot studies (first stage of research):

- after 24 h of storage at 5 °C.

For main research (second stage of research):

- after 24 h of storage at 5 °C.
- after 7 days of storage at 5 °C.
- after 14 days of storage at 5 °C.
- after 21 days of storage at 5 °C.

#### 2.2.4. Number of Yogurt Bacteria Analysis

Microbiological analysis was performed in Petri dishes. The M17 agar medium (Merck, Darmstadt, Germany) was used to determine the number of *S. thermophilus*, while MRS (De Man, Rogosa and Sharpe) agar medium (Merck, Germany) was used to determine the number of *L. delbrueckii* subsp. *bulgaricus*. The media were prepared and sterilized (M17 agar: 121 °C, 15 min; MRS agar: 117 °C, 15 min) several days prior to inoculation. Petri dishes with media were incubated at 37 °C for 2 days to dry them and then stored at 5 °C until analysis.

The drop plate method was used to determine the number of yogurt bacteria cells. A dilution series of test samples was prepared ranging from 10^−1^ to 10^−6^. The drop in inoculation was carried out for dilutions ranging from 10^−6^ to 10^−3^ by placing 20 μL of each dilution in appropriately marked zones of the plates. The M17 agar plates were incubated at 37 °C for 48 h under aerobic conditions, while the MRS agar plates were incubated at 37 °C for 72 h under anaerobic conditions. Anaerobic cultures were obtained in Anaerocult containers (Merck, Germany). After incubation, the grown colonies were counted and converted to CFU/mL. The result was expressed as the logarithm of the total cell count of *S. thermophilus* and *L. delbrueckii* subsp. *bulgaricus*. The incubation was performed in 3 replications according to the following measurement points:

- after 24 h of storage at 5 °C.
- after 7 days of storage at 5 °C.
- after 14 days of storage at 5 °C.
- after 21 days of storage at 5 °C.

#### 2.2.5. Statistical Analysis

The results of the first stage of the study were subjected to one-way analysis of variance (ANOVA), and the results of the second stage of the study were subjected to two-way ANOVA using the software Statistica 13.1 (StatSoft, Kraków, Poland). The two-way ANOVA allowed us to determine the effect of the storage period and the form of flaxseed supplementation and their interaction on the studied quality characteristics of yogurt. The significance of the differences was analyzed by Tukey’s test at α = 0.05. Results are presented as mean and standard deviation (SD).

## 3. Results

### 3.1. Pilot Study Results

In the first stage of the research, four variants of yogurt were prepared to determine the optimal dose of flaxseed supplement. Milled flaxseed was used in three doses (1%, 2%, and 3%), which were added before yogurt fermentation. The type and dose of the supplement are important in the parameters of finished products. These parameters include active acidity, apparent viscosity, and syneresis. The addition of flaxseed to yogurt at levels of 1%, 2% and 3% was selected based on the study by Kumar et al. [35], which investigated the effect of the addition of flaxseed oil, flaxseed flour and fruits for the sensory, physico-chemical, and fatty acid profile of yogurt. The study showed that fruit yogurt (20% fruit and sugar mixture) with the incorporation of flaxseed oil up to 2% and flaxseed flour up to 1% in combination can be used for the preparation of fruit yogurt with acceptable sensory attributes. The scores drastically reduced for yogurt samples wherein 2% flaxseed flour was incorporated. The results indicate that too high a dose of flaxseed addition to yogurt may have a negative impact on the sensory acceptability of the product.

To determine the effect of the addition of various doses of milled flaxseed on acidity, the pH of the obtained yogurts was analyzed after 3 h of incubation at 37 °C and 24 h of storage at 5 °C (Table 1). The results obtained revealed that the addition of milled flaxseed, regardless of the dose used, did not significantly affect the pH of the yogurts obtained as compared to that of the control sample, both after 3 h of incubation and 24 h of storage.

The apparent viscosity analysis showed that the addition of milled flaxseed to yogurts in the amount of 1% significantly increased the apparent viscosity in the tested yogurts as compared to that of the control sample (Table 1). The addition of milled flaxseed in the amount of 2% and 3% significantly reduced the viscosity of the tested yogurts as compared to that of the control sample. With an increase in the amount of the added flaxseed, the apparent viscosity in the tested yogurts decreased significantly.

The analysis of syneresis showed that the addition of milled flaxseed to yogurts in the amount of 1% did not significantly affect the syneresis in the tested yogurts as compared to that in the control sample (Table 1). For higher doses of flaxseed (2% and 3%), a significant increase in the syneresis was observed in the tested yogurts as compared to that in the control sample.

The optimal dose of milled flaxseed supplement was 1% relative to the amount of milk. The addition of flaxseed in this amount allowed yogurts to be obtained with the lowest degree of whey leakage (syneresis) and the highest apparent viscosity. These parameters allowed a product to be obtained with properties that were almost like those of basic yogurt without additives (control sample).

### 3.2. Main Research Results

#### 3.2.1. Active Acidity (pH) Analysis

During the refrigerated storage of yogurts, changes in active acidity may occur, which affect the organoleptic properties of the product. It is desirable to maintain the acidity of the product during the storage period at a level close to the initial acidity. In the yogurts tested, changes in active acidity during storage were examined by determining it after the fermentation process, and after 7, 14, and 21 days of storage (Table 2).

The analysis showed that time of storage (*p* < 0.001) and form of flaxseed supplementation (*p* = 0.006) significantly influenced the active acidity in the tested yogurts. There was no significant interaction effect of the storage time × form of flaxseed supplementation for active acidity of the tested yogurts (*p* = 0.959). In all samples, the acidity changed significantly after 7 days of storage. There was a general tendency of decrease in the pH of the tested yogurts, both in the control sample and in the variants with the addition of milled and whole flaxseed. A significantly lower decrease in pH as compared to other samples was demonstrated for the addition of milled flaxseed added after the fermentation process.

#### 3.2.2. Apparent Viscosity Analysis

To assess whether the addition of flaxseed affects the natural stabilization of the yogurts obtained, their apparent viscosity was analyzed. In the tested yogurts, changes in apparent viscosity during storage were analyzed by determining the viscosity after the fermentation process and after 7, 14, and 21 days of storage (Table 2). The conducted analysis showed that time of storage (*p* < 0.001) and form of flaxseed supplementation (*p* < 0.001) significantly influenced the changes in apparent viscosity in the tested yogurts. There was a significant interaction effect of the storage time × form of flaxseed supplementation for apparent viscosity of the tested yogurts (*p* < 0.001). The changes in the apparent viscosity of the yoghurts were already apparent after 7 days of storage. The ANOVA results showed a statistically significant increase in viscosity for yogurt with milled flaxseed added before fermentation and for yogurts with milled and whole flaxseed added after fermentation as compared to that for the control sample. The highest viscosity was shown by yogurt with milled flaxseed added after fermentation, while the lowest one was shown by yogurt with whole flaxseed added before fermentation.

#### 3.2.3. Syneresis Analysis

To determine whether the addition of flaxseed as a natural stabilizer would improve the quality of the tested yogurts, syneresis was assessed, which indicates the degree of leakage of whey from the yogurt. The changes in syneresis in the yogurts during the storage period were determined after the fermentation process and after 7, 14, and 21 days of storage (Table 2). The analysis showed that time of storage (*p* < 0.001) and form of flaxseed supplementation (*p* < 0.001) significantly influenced the apparent viscosity in the tested yogurts. There was significant interaction effect of the storage time × form of flaxseed supplementation for syneresis of the tested yogurts (*p* < 0.038). The analysis of variance showed that time of storage had a significant effect on syneresis only for yogurts with the addition of whole flaxseed, both before fermentation (Variant 2) and after fermentation (Variant 4). For these yogurts, syneresis was significantly increased after 7 days of refrigerated storage. In the remaining samples, syneresis was at a similar level during the entire storage period.

The analysis conducted showed that the form of adding flaxseed to yogurts significantly influenced their syneresis. The highest ability to retain whey (the lowest syneresis) was shown by yogurt with milled flaxseed added after fermentation. The lowest ability to retain whey (the highest syneresis) was shown by yogurt without the addition of flaxseed (control sample) and by yogurt with whole flaxseed added before fermentation.

#### 3.2.4. Analysis of Number of Yogurt Bacteria

In the yogurts tested, the number of selected LAB was analyzed to determine whether their number is typical for yogurts available on the market. According to the International Dairy Federation [36], the number of viable LAB cells in yogurt should not be less than 7 log CFU/mL. The changes in the number of *S. thermophilus* and *L. delbrueckii* subsp. *bulgaricus* cells in the tested yogurts during the storage period were determined after the fermentation process and after 7, 14, and 21 days of storage (Table 2). Before fermentation, the number of yogurt bacteria in the tested samples was 8.5 log CFU/mL, and after the fermentation process, the total number of yogurt bacteria of the obtained yogurts was 8.6–9.1 log CFU/mL.

The analysis showed that time of storage (*p* < 0.929) and form of flaxseed supplementation (*p* < 0.734) did not significantly affect the changes in the number of yogurt bacteria in the tested yogurts. There was no significant interaction effect of the storage time × form of flaxseed supplementation for the number of yogurt bacteria of the tested yogurts (*p* = 0.969). After fermentation and during 21 days of storage, the number of tested LAB in all types of yogurts was at a similar level, i.e., all obtained yogurts had a normative number of viable cells (≥10^6^ CFU/mL or g). The analysis showed no statistically significant differences between the number of yogurt bacteria in the tested yogurts. This value was similar in all the tested samples, which indicates that the addition of flaxseed did not significantly change the content of yogurt bacteria.

The mean number of *S. thermophilus* and *L. delbrueckii* subsp. *bulgaricus* cells for all the tested yogurts was 8.66 and 8.4 log CFU/mL, respectively, and did not differ significantly during the entire storage period. Therefore, the tested yogurts showed microbial stability in terms of count of lactobacilli and streptococci during the entire storage process.

## 4. Discussion

The pilot study showed that the addition of milled flaxseed to yogurts in the amount of 1% relative to the amount of milk was favorable. The use of this dose allowed yogurt to be obtained with the lowest syneresis, which indicates the amount of whey leakage. A similar tendency was found for the addition of selected hydrocolloids to yogurt [37]. The lowest syneresis was obtained in yogurts with the addition of carrageenan, locust bean gum, guar gum, and xanthan gum (0.01%), and syneresis became higher with the increase in their concentration. This effect may be because the casein micelles were surrounded by the stabilizer molecules, which reduced the interactions between the casein micelles. The use of a 1% dose of milled flaxseed in the pilot study also allowed yogurts to be obtained with the highest apparent viscosity. The presence of natural additives in dairy products, which have a positive effect on their apparent viscosity, also influences their better acceptance among consumers [38].

Similar results were obtained by Ismail et al. [39], who studied the influence of milk supplemented with different percentages of flaxseed oil (0.0%, 0.5%, 1.0%, 1.5%, and 2.0%) on the physicochemical, microbiological, and organoleptic characteristics of yogurt during storage at 6 °C for 14 days. Yogurt samples supplemented with a different percentage of flaxseed oil showed higher curd tension and whey syneresis than the control sample. The supplementation of milk with 1.0% flaxseed oil was found to be most acceptable.

Mousavi et al. [40] produced prebiotic yogurt supplemented with powdered flaxseed (0–4%) and investigated its texture and sensory properties; however, they obtained a different conclusion. The addition of flaxseed to yogurt samples increased the hardness, gumminess, chewiness, cohesiveness, and springiness values of the produced yogurt samples. The addition of 2.63% flaxseed into yogurt samples enables functional food to be produced with satisfactory texture and sensory characteristics. The differences may be due to the origin of the tested flaxseed (Poland vs. Iran), as the composition of flaxseed can vary with genetics, growth environment, seed processing, and analytical method [18].

In the main study, an optimized dose of flaxseed (1%) was used, and it was assessed whether the selected qualities of yogurt were affected by the form of flaxseed (whole or milled) and by the time of addition (before or after fermentation). The study conducted showed that time significantly influenced the active acidity, apparent viscosity, and syneresis in the tested yogurts. Active acidity (pH) decreased significantly after 7 days of storage. The study showed that the form of flaxseed supplement significantly influences the pH of the tested yoghurts. A significantly lower decrease in pH as compared to other samples was demonstrated for the addition of milled flaxseed added after the fermentation process. However, for all samples, the acidity did not decrease below 4.19 during the entire storage period, which is within the normal yogurt pH range of 4.0 to 4.5 [9]. Similar results were obtained by Jeong et al. [41], who studied the physicochemical characteristics of bioactive kefir containing different concentrations of flaxseed. The pH of this bioactive kefir decreased with increasing incubation time, which lasted for 48 h. The final pH value was 4.50, 4.52, and 4.51, respectively, for kefir with the addition of 1%, 2%, and 3% flaxseed. However, these parameters were not affected by the amount of added flaxseed.

The significant increase in the viscosity of the tested yogurts with time was demonstrated for yogurt containing milled flaxseed added before fermentation and for yogurt containing milled and whole flaxseed added after fermentation in relation to the control sample. The quality of the raw material, the type of structure-forming additives, the type of microorganisms, and the fermentation conditions affect the rheological properties of the yogurt. It is necessary to obtain the desired viscosity of the yogurt that remains throughout its shelf life [42]. The highest viscosity was possessed by yogurt with milled flaxseed added after fermentation, and the lowest by yogurt with whole flaxseed added before fermentation. It can therefore be concluded that the addition of milled flaxseed has a positive effect on the apparent viscosity of the obtained yogurts, regardless of whether it is added before or after the fermentation process. To obtain yogurt with the addition of whole flaxseed with increased apparent viscosity, it is necessary to add whole flaxseed after the fermentation process. Similar results were obtained by Marand et al. [43], who tested yogurt samples enriched with flaxseed powder. The results showed that the addition of flaxseed powder significantly increased water holding capacity and the viscosity of yogurts.

Time significantly affects the increase in syneresis for yogurts with whole flaxseed added before or after fermentation. In other samples, syneresis remained at a constant level during the entire period of storage. The highest ability to retain whey (the lowest degree of syneresis) was found in yogurt with milled flaxseed added after fermentation. The lowest ability to retain whey (the highest degree of syneresis) was found in yogurt without flaxseed (control sample) and by yogurt with whole flaxseed added before fermentation. Therefore, it can be concluded that to reduce syneresis in yogurts, the addition of milled flaxseed (both before and after fermentation) is more advantageous, because it did not significantly affect the increase in the leakage of whey from the products.

Leakage of whey can limit the shelf life of yogurts. To prevent this leakage, it is common to use stabilizers such as pectin, carrageenan, guar gum, starch, or locust bean gum. The addition of stabilizers also serves to reduce the calorie count of yogurt by limiting the fat content and makes the yogurt economically cheaper by reducing the consumption of skim milk powder [34,44]. Reduced syneresis in yogurts with milled flaxseed may result from the presence of mucilage polysaccharides, which can bind water and are released during the grinding process [45]. Arabshahi-Delouee et al. [46] studied the effect of the addition of flaxseed mucilage on the physicochemical and sensorial properties of semi-fat (1.5% fat) set yogurt. Yogurt samples were incorporated with flaxseed mucilage at the levels of 0.00%, 0.10%, 0.15%, and 0.20% and analyzed periodically during storage at 5 °C (1, 7, and 15 days). The pH value of all the samples significantly decreased; viscosity, consistency, and water holding capacity increased; and the syneresis value decreased with the increase in the amount of flaxseed mucilage and storage time.

Time and form of flaxseed supplementation did not significantly affect the changes in the number of yogurt bacteria in the tested yogurts. No significant differences were observed between the number of yogurt bacteria in the tested yogurts, which indicates that the addition of flaxseed did not significantly change the content of yogurt bacteria. The average number of yogurt bacteria of the species *S. thermophilus* for all the tested yogurts was 8.6 log CFU/mL and that of *L. delbrueckii* subsp. *bulgaricus* was 8.4 log CFU/mL, and these values did not differ significantly throughout the storage period. Mihoubi et al. [47] used two types of milk powder for manufacturing yogurt: skimmed milk powder at 15% (*w*/*v*) and whole milk powder at 13.7% (*w*/*v*), which were fermented and stored at 4 °C for 28 days. The addition of ground flaxseed decreased the pH values during fermentation and refrigerated storage. In the yogurts obtained, the average values of LAB CFU counts for the control sample were significantly increased (*S. thermophilus*: 9.2 log CFU/mL; *L. bulgaricus*: 8.7 log CFU/mL at the 14th day of storage); however, a reduction was noted during the last two weeks of storage (*S. thermophilus*: 8.1 log CFU/mL; *L. bulgaricus*: 6.9 log CFU/mL). This effect was attributed to *L. delbrueckii* subsp. *bulgaricus*, which produces lactic acid during cold storage. This process is known as post-acidification in the dairy industry. Lactic acid produced during cold storage causes loss of bacterial viability.

In the tested yogurts, slight differences were noted between the amount of *Lactobacillus* and *Streptococcus*, with a slight dominance of *S. thermophilus*. These differences were not more than 1 log unit. The pH value showed that the acidity did not drop below 4.0. This may be due to the smaller population of *L. delbrueckii* subsp. *bulgaricus*, which has a greater acidifying capacity than lactic streptococci. This bacterial species can lower the pH value to 3.8. *S. thermophilus* is not resistant to high acidity. Its development is inhibited at pH 4.1 [48]. A comparable relationship was found in studies that analyzed the microflora of yogurts following the addition of amaranth seeds and oat grains [36]. The tested yogurts showed a lower level of *L. delbrueckii* subsp. *bulgaricus* than that of *S. thermophilus*. As noted for yogurts with the addition of flaxseed, throughout the cold storage period, yogurts with the addition of amaranth and oats showed a high level of characteristic microflora in accordance with the FIL/IDF (Fédération Internationale de Laitiere/International Dairy Federation) guidelines. The present study showed that the addition of flaxseed has a positive effect on the selected quality characteristics of yogurt. The results indicate that to achieve increased apparent viscosity and to reduce syneresis, it is more advantageous to use milled flaxseed rather than whole flaxseed.

The 1% addition of flaxseed allows yogurts to be obtained with favorable technological parameters. However, such an addition is probably insufficient to provide a therapeutic effect, e.g., for the prevention of cardiac diseases. Previous studies indicate that a daily intake of about 20–40 g of flaxseed has a positive effect on health by improving the lipid profile and acting against breast cancer [49,50]. Yogurts are usually available on the market in packages weighing 150–250 g. Yogurt with this weight containing 1% of flaxseed will allow 1.5–2.5 g of this ingredient in one package. This amount of flaxseed has a positive effect on the selected physico-chemical properties of the obtained yogurts and can be just one of the sources of this component during the day. Increasing the dose of flaxseed in yogurt could require the use of additional stabilizers in its production, which would make it impossible to obtain a “clean label” product. Future research should focus on obtaining yogurts with a dose of linseed close to the therapeutic dose, which also have good physico-chemical properties. The presented study focused mainly on the effect of the addition of different doses of flaxseed on the physico-chemical properties of yogurt—mainly its stability. However, there is a need for further research to determine the impact of the storage process on the chemical composition of flaxseed. The impact of the storage process on the level and bioavailability of selected beneficial compounds of flaxseed, such as ALA and soluble fiber, should also be investigated. This will allow the potential impact of the consumption of yogurt with the addition of flaxseed on the improvement of human health to be determined.

## 5. Conclusions

The pilot study showed that the optimal dose of flaxseed added to the tested yogurts was 1% relative to the amount of milk. The main study showed that the time and form of flaxseed supplementation significantly influenced the active acidity, apparent viscosity, and syneresis in the tested yogurts. Active acidity (pH) decreased significantly after the 7 days of storage, and a significantly lower decrease in pH as compared to other samples was demonstrated for the addition of milled flaxseed added after the fermentation process. The highest viscosity was found in yogurt with milled flaxseed added after fermentation, and the lowest viscosity was found in yogurt with whole flaxseed added before fermentation. Therefore, it can be concluded that the addition of milled flaxseed has a positive effect on the apparent viscosity of the yogurts obtained, regardless of whether it is added before or after the fermentation process. Time significantly affects the increase in syneresis for yogurts with added whole flaxseed—both before or after fermentation. Therefore, it can be concluded that to reduce syneresis in yogurts, the addition of milled flaxseed (both before and after fermentation) is more advantageous, because it does not significantly affect the increase in the leakage of whey from the products. No significant differences were observed between the number of yogurt bacteria in the tested yogurts, which indicates that the addition of flaxseed in any form did not significantly change the content of yogurt bacteria.

The present study shows that the addition of flaxseed has a positive effect on the selected quality characteristics of yogurt. The results indicate that to achieve increased apparent viscosity and to reduce syneresis, it is more advantageous to use milled flaxseed rather than whole flaxseed. The functional and health-promoting properties of flaxseed can help the food processing industry to create a greater variety of foods that contain this plant.

## Figures and Tables

**Table 1:** Assessment of the quality parameters of yogurts with various additives of milled flaxseed.

Yogurt	pH after 3 h of Incubation	pH after 24 h of Storage	Apparent Viscosity [mPa × s]	Syneresis [%]
flaxseed 0%	4.76 ± 0.01 ^a^	4.36 ± 0.32 ^a^	6312.00 ± 9.85 ^a^	29.90 ± 2.31 ^a^
flaxseed 1%	4.43 ± 0.26 ^a^	4.25 ± 0.32 ^a^	9498.00 ± 79.90 ^b^	26.10 ± 4.43 ^a^
flaxseed 2%	4.51 ± 0.71 ^a^	4.32 ± 0.94 ^a^	1308.00 ± 17.08 ^c^	51.10 ± 7.63 ^b^
flaxseed 3%	4.61 ± 0.80 ^a^	4.43 ± 0.58 ^a^	4408.00 ± 68.53 ^d^	43.10 ± 2.00 ^b^

Table shows mean values ± standard deviations; a, b, c, d-mean values in columns denoted by different letters differ significantly (*p* ≤ 0.05).

**Table 2:** Changes in quality characteristics during the storage period of the tested yogurts.

Quality Characteristics	Type of Sample	Storage Time [day]			
		1	7	14	21
pH	Control sample	4.42 ± 0.05 ^a^	4.34 ± 0.04 ^b^	4.27 ± 0.09 ^b^	4.20 ± 0.06 ^c^
	Variant 1	4.49 ± 0.08 ^a^	4.38 ± 0.09 ^b^	4.27 ± 0.02 ^c^	4.22 ± 0.07 ^c^
	Variant 2	4.44 ± 0.10 ^a^	4.36 ± 0.02 ^b^	4.29 ± 0.09 ^b^	4.22 ± 0.06 ^c^
	Variant 3	4.57 ± 0.10 ^a^	4.47 ± 0.06 ^b^	4.32 ± 0.01 ^c^	4.31 ± 0.06 ^c^
	Variant 4	4.47 ± 0.09 ^a^	4.37 ± 0.08 ^b^	4.28 ± 0.03 ^b^	4.19 ± 0.06 ^c^
apparent viscosity [mPa×s]	Control sample	1950.00 ± 29.02 ^a^	2580.00 ± 7.00 ^b^	2742.00 ± 14.64 ^c^	2782.00 ± 31.43 ^c^
	Variant 1	4764.00 ± 24.84 ^a^	5064.00 ± 7.55 ^b^	4596.00 ± 21.57 ^c^	5332.00 ± 13.05 ^d^
	Variant 2	1124.00 ± 16.65 ^a^	1682.00 ± 9.94 ^b^	2522.00 ± 26.65 ^c^	2294.00 ± 22.94 ^d^
	Variant 3	6624.00 ± 5.13 ^a^	5126.00 ± 22.87 ^b^	5470.00 ± 18.93 ^c^	5456.00 ± 21.22 ^c^
	Variant 4	4684.00 ± 6.66 ^a^	5086.00 ± 17.58 ^b^	4702.00 ± 15.27 ^a^	4202.00 ± 25.01 ^c^
syneresis [%]	Control sample	40.60 ± 9.46 ^a^	50.10 ± 9.05 ^a^	52.40 ± 10.91 ^a^	44.70 ± 11.55 ^a^
	Variant 1	36.40 ± 9.37 ^a^	43.60 ± 6.41 ^a^	47.90 ± 5.62 ^a^	42.10 ± 11.19 ^a^
	Variant 2	26.20 ± 4.91 ^a^	44.20 ± 9.60 ^b^	58.70 ± 5.64 ^b^	46.40 ± 5.72 ^b^
	Variant 3	20.50 ± 5.76 ^a^	25.60 ± 5.68 ^a^	24.10 ± 6.93 ^a^	17.00 ± 3.00 ^a^
	Variant 4	20.10 ± 5.43 ^a^	50.10 ± 5.98 ^b^	52.40 ± 5.25 ^b^	44.70 ± 6.64 ^b^
number of yogurt bacteria [log CFU/mL]	Control sample	8.90 ± 0.21 ^a^	9.00 ± 0.42 ^a^	9.00 ± 0.13 ^a^	9.00 ± 0.53 ^a^
	Variant 1	8.90 ± 0.44 ^a^	8.70 ± 0.45 ^a^	9.00 ± 0.46 ^a^	9.10 ± 0.99 ^a^
	Variant 2	9.00 ± 0.78 ^a^	9.00 ± 0.44 ^a^	9.00 ± 0.45 ^a^	8.80 ± 0.53 ^a^
	Variant 3	8.80 ± 0.62 ^a^	8.70 ± 0.26 ^a^	8.70 ± 0.52 ^a^	8.80 ± 0.62 ^a^
	Variant 4	8.90 ± 1.01 ^a^	8.80 ± 0.61 ^a^	8.60 ± 0.26 ^a^	8.60 ± 0.61 ^a^

Control sample—yogurt without the addition of whole or milled flaxseed; Variant 1—yogurt with milled flaxseed added before fermentation; Variant 2—yogurt with whole flaxseed added before fermentation; Variant 3—yogurt with milled flaxseed added after fermentation; Variant 4—yogurt with whole flaxseed added after fermentation. Table shows mean values ± standard deviations; a, b, c, d-mean values in rows denoted by different letters differ significantly (*p* ≤ 0.05).

## Data Availability

The datasets generated for this study are available on request to the corresponding author.
